# Supplementary material for: The Physics behind the Modulation of Thermionic Current in Photodetectors Based on Graphene Embedded between Amorphous and Crystalline Silicon
Source: Nanomaterials (Basel). 2023 Feb 26;13(5):872. doi: 10.3390/nano13050872 (PMC10005663; doi:10.3390/nano13050872)
Supplement: Supplementary file 1 [file nanomaterials-13-00872-s001.zip › nanomaterials-2211326-supplementary.pdf]

nanomaterials

Supplementary Materials

The Physics behind the Modulation of Thermionic Current in Photodetectors Based on Graphene Embedded between Amorphous and Crystalline Silicon

Teresa Crisci [1,2], Piera Maccagnani [3,4,*], Luigi Moretti [2], Caterina Summonte [3], Mariano Gioffrè [1], Rita Rizzoli [3] and Maurizio Casalino [1]

1 Institute of Applied Science and Intelligent Systems "Eduardo Caianiello" (CNR), 80131 Napoli, Italy
2 Department of Mathematics and Physics, University of Campania "Luigi Vanvitelli", 81100 Caserta, Italy
3 Institute for Microelectronics and Microsystems (CNR), 40129 Bologna, Italy
4 Department of Physics and Earth Sciences, University of Ferrara, Via Giuseppe Saragat 1/c, 44122 Ferrara, Italy
* Correspondence: maccagnani@bo.imm.cnr.it

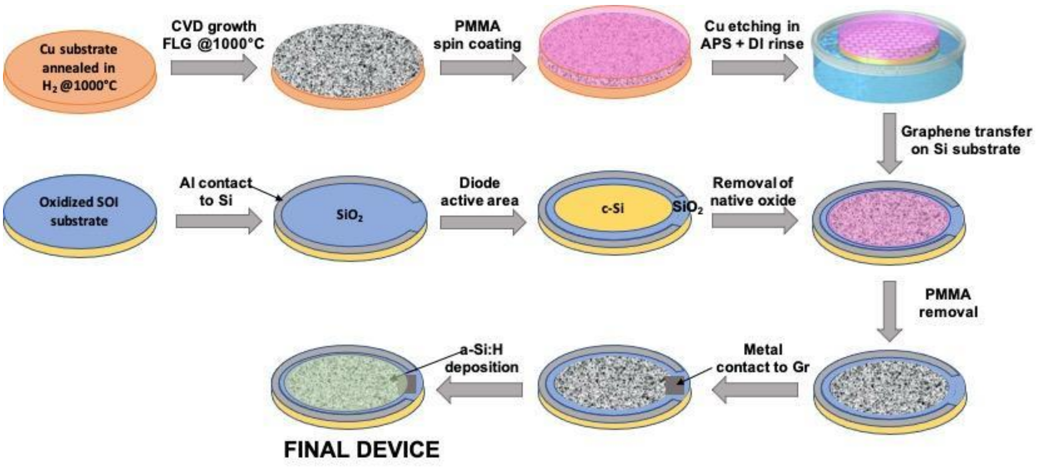

Figure S1. Scheme of the fabrication procedure for the photodetector (as described in Section 3.1). The graphene layer is embedded between amorphous (a-Si:H) and crystalline silicon (c-Si).

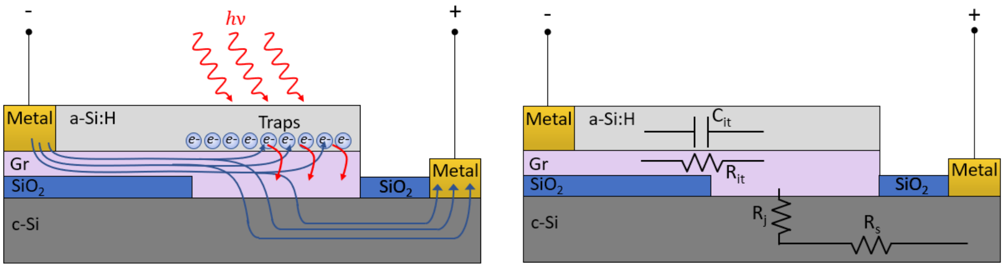

(a) (b)

Figure S2. (**a**) Sketch of the device showing the path of carriers moving from the graphene contact towards the aluminum electrode, under a negative voltage applied to graphene. Blue arrows represent current flow associated with the applied bias, while red arrows represent the carriers released from traps under NIR illumination. (**b**) Electronic components associated with the proposed device and schematized in Figure 7.

Working in the NIR (around 1550 nm), the refractive index of graphene reported in Eq. 1 doesn't significantly change moving from 1 layer to 4 layers. Given that the absorption of a material is proportional to the product of the absorption coefficient times the thickness, an unchanged absorption coefficient for FLG with respect to SLG is in agreement with the largely reported result that the optical absorption of graphene increases additively by increasing the number of layers.

To verify the application of Equation 1 even in the case of multiple layers, we carried out numerical simulations of suspended graphene with thicknesses: 0.335 nm (1 layer), 0.67 nm (2 layers), 1.005 nm (3 layers) and 1.34 nm (4 layers). Numerical results reported in Fig. S3 are close to what we expect from theory (additive effect of the optical absorption by increasing the number of layers).

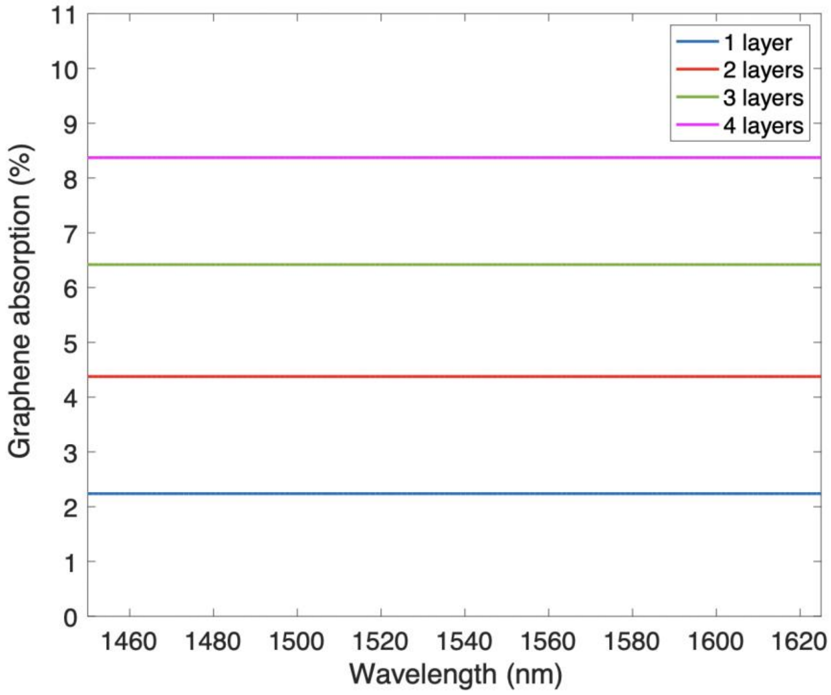

Figure S3. Numerical simulation of graphene optical absorption by varying the number of graphene layers from 1- to 4 -layers, obtained using Equation 1 reported in the manuscript.
